# ENGAGING COMMUNITIES TO STRENGTHEN RESEARCH ETHICS IN LOW-INCOME SETTINGS: SELECTION AND PERCEPTIONS OF MEMBERS OF A NETWORK OF REPRESENTATIVES IN COASTAL KENYA

**DOI:** 10.1111/dewb.12014

**Published:** 2013-02-21

**Authors:** Dorcas M Kamuya, Vicki Marsh, Francis K Kombe, P Wenzel Geissler, Sassy C Molyneux

**Keywords:** research ethics, community network, community engagement, developing countries, representation

## Abstract

There is wide agreement that community engagement is important for many research types and settings, often including interaction with ‘representatives’ of communities. There is relatively little published experience of community engagement in international research settings, with available information focusing on Community Advisory Boards or Groups (CAB/CAGs), or variants of these, where CAB/G members often advise researchers *on behalf of* the communities they represent. In this paper we describe a network of community members (‘KEMRI Community Representatives’, or ‘KCRs’) linked to a large multi-disciplinary research programme on the Kenyan Coast. Unlike many CAB/Gs, the intention with the KCR network has evolved to be for members to represent the geographical areas in which a diverse range of health studies are conducted *through being typical* of those communities. We draw on routine reports, self-administered questionnaires and interviews to: 1) document how typical KCR members are of the local communities in terms of basic characteristics, and 2) explore KCR's perceptions of their roles, and of the benefits and challenges of undertaking these roles. We conclude that this evolving network is a potentially valuable way of strengthening interactions between a research institution and a local geographic community, through contributing to meeting intrinsic ethical values such as showing respect, and instrumental values such as improving consent processes. However, there are numerous challenges involved. Other ways of interacting with members of local communities, including community leaders, and the most vulnerable groups least likely to be vocal in representative groups, have always been, and remain, essential.

## Introduction

### Community engagement and identifying community representatives

There is wide agreement on the importance of community engagement (CE) in many areas of research and types of research settings. There is particular interest in CE in international research settings, where there are often big differences between researchers and typical participant communities in social and cultural norms, values, goals, resources and in levels of technological understanding.^1^ However, it is also recognised that CE may sometimes not be appropriate, especially where it has potential to further stigmatize particular groups of communities.^2^

Arguments for strengthening CE in international research include to:

- Identify and minimise ‘internal risks’ (those only visible to those within a community), such as social identity and equilibrium,^3^ and risks that can be imposed externally such as stigmatization of the community.^4^
- Strengthen informed consent processes in research through dissemination of information on research goals, risks and benefits, and incorporating local views into consent processes.^5^
- Empower communities and demonstrate respect, both as a goal in itself and to, in turn, strengthen mutual understanding, trust and credibility of researchers.^6^
- Strengthen the acceptability and quality of research.

Engagement with communities often involves interacting with either ‘the general community’ (however defined) or with some selected members or ‘representatives’ of those communities. The community representatives interface with research staff and can potentially be involved in a broad range of research activities, including protocol development, providing information and obtaining consent, data collection, reviewing access to data and samples, and dissemination or as co-authors in publication of research findings.

There is relatively little published experience of CE in practice in international research settings. The information that is available is focused on Community Advisory Boards or Groups (CAB/CAGs), or variants of these, not least because such groups are increasingly recommended or even required by research funders.^7^ In many drug or vaccine trials, establishment of CAB/Gs has therefore come to be understood as ‘standard practice’.^8^

Studies to date suggest that two key challenges in CE include defining the communities of interest in research, and identifying who can be considered to ‘authentically’ represent those communities.^9^ In broad terms, definitions of community can be based on geography, on special interests or goals, or shared situations or experiences.^10^ For individuals themselves, community membership may be choice-based (for example membership of a women's group), or linked to innate personal characteristics (such as age or ethnic group). In many cases people are members of multiple communities, with membership shifting over time and space. In health research more specifically, definitions of community can be internally or externally defined, but in much non-participatory research, relevant communities at least initially are often defined by researchers who are external to communities.^11^ Definitions of communities are therefore often related to the nature of the research activity (for example, does it involve a particular geographical area or illness group) and where the institution is based (for example, is it based in a rural or urban setting). For CAB/Gs, often initiated by researchers working in low income settings^12^ varying definitions of community have contributed to some groups including members from a broad cross section of the community (‘*broad community*’, see for example Shubis et al.),^13^ and others consisting more of a particular population identified in a proposal (‘*populations specific*’, see for example Morin et al.).^14^

In terms of selection of representatives of the communities that are identified either internally or (more typically) externally in CE activities, individuals might be selected or select themselves to *speak on behalf* of a particular community. They might also be selected or select themselves as typical members of that community; where their views reflect those of their communities through being typical of other community members. Individuals considered typical might be identified on the basis of characteristics such as where they live, their education level or their religion. Representatives who speak on behalf of communities are often relatively charismatic, well known, and outspoken, such as leaders of large women's groups or religious elders.^15^ These characteristics may make these representatives more able to voice their views and options, and ensure they are heard, but it may also mean they are rather unusual or express atypical ideas or approaches. Typical community members may be less well known and vocal, but may have greater contact with and awareness of everyday issues and concerns in their communities, including those of the most vulnerable and marginalized members.

Although CAB/Gs vary significantly across and within low income sites, in many cases members appear to be asked to advise researchers on behalf of the communities they represent, as opposed to through being typical members of those communities. This is not always openly stated, but is suggested through selection procedures that include requiring members to be literate, and asking organisations such as women's groups to nominate or elect a member. It may also be suggested through roles that require members to act as a ‘bridge’ between community members and researchers, or to raise issues and make recommendations ‘on behalf of’ community members.

While working through CAB/Gs has been shown to strengthen research relationships and ethical practice,^16^ documented challenges beyond defining communities and their representatives, as described above, have included ensuring clarity in roles and adequate training to fulfil those roles, facilitating appropriate selection and motivation of members, and avoiding politicisation. A specific set of tensions have been identified around the dual functions that some CAB/Gs have of both advancing the research and protecting the community;^17^ duel functions that can potentially conflict with one another.

In this paper we contribute to the small but growing body of work documenting experience of working with community representatives in low income settings by describing and evaluating the establishment of a network of representatives which was set-up by a large biomedical research programme. Drawing on the above distinctions from the literature, we aimed to establish a network of representatives of the broad communities in the geographical area within which much of the research takes place. Over time we have shifted our approach from identifying representatives who speak ‘on behalf of’ local community members towards those who are more ‘typical’. The network of KEMRI community representatives was established as an additional channel of interaction beyond the more formally recognised community leaders and gate-keepers such as chiefs, village elders and other opinion leaders, and beyond the institution's more population specific CAB for HIV studies. The entire set of community engagement activities is an action research programme where we are continuously acting, learning, changing and re-acting.

In a previous paper we shared some initial views on the strengths and challenges of working with this network of representatives.^18^ At that stage we were not in a position to share the views and insights of the KCRs themselves. In this paper we describe the institutional context and the approach to selecting KCRs, and the study methods. We then present the demographic characteristics of KCRs and compare these with those of the population they represent, and explore KCRs own perceptions of their roles, and of the challenges and benefits of undertaking those roles. We conclude by showing how we have drawn upon these data and on broader experiences to amend the selection of the latest network of KCRs in an effort to ensure that they are more typical members of the area, a feature we are increasingly emphasising.

### Establishing a network of community representatives at the KEMRI-Wellcome Trust Programme

The Kenya Medical Research Institute (KEMRI) Centre for Geographical Medicine Research, Coast (CGMRC) is one of 10 research centres in Kenya administered by KEMRI, a parastatal organisation under the Ministry of Health, and mandated to carry out health research in Kenya. A collaborative research programme was set up between KEMRI CGMRC^19^ and the Wellcome Trust in Kilifi in 1989. A diverse range of health research activities, from basic immunology through clinical studies to implementation studies, are conducted by the programme across East Africa. Some studies draw on the KDHSS (Kilifi Demographic and Health Surveillance System); a geographical area covering about 240,000 people in 5 government administrative ‘locations’ surrounding Kilifi District Hospital (see Scott et al.^20^ for current figures).

The KCR network was set up as one activity amongst many in a broader community engagement programme for the institution. The intention behind selection processes for KCRs was to enable residents of the locations to assist with the selection of representatives, and to ensure that those representatives came from and were aware of ideas and concerns across the geographical area. In this way, the representatives would be physically located across a wide area, and be accepted by people from their location as appropriate to interact with KEMRI.

The process of selecting and working with this new group of KCRs in 11 locations has been described in detail elsewhere,^21^ and is summarised in Box 1. Steps 1 to 3 apply to new KCRs. In summary, two selection methods were used; in one method used in 3 locations, area administrative leaders (chiefs) nominated the prospective representatives; in the other method used in 11 locations, local Community Based Organizations (CBO)^22^ nominated their own members as representatives. The CBO system was used as members are local residents, meet regularly, and are a recognised existing community communication channel.

**Box 1. KCR selection and meeting processes**

1. Formative research identified formal and informal community based organizations (CBOs) across the KHDSS area,
2. Requested the CBOs from a given location to send two representatives to an open day at the research centre; several representatives attended open day from each location.
3. Locational CBO representatives at the open day nominated KCR members.
4. Nominated KCRs trained on basics of research and research ethics using participatory approaches.
5. Public endorsement meetings held at each location for nominated KCRs in the locations they represented; 12 KCR group members endorsed, 2 rejected and re-election held.
6. KCR groups elected chairperson, vice chairperson and secretary; and jointly with CLG members defined their terms of reference
7. Quarterly and ad hoc location-based meetings held between KCR location groups and community liaison staff from the research programme; meetings were chaired by KCR chairperson and supported by KEMRI with bus fare refunds - $4.3 per person per meeting – and stationary. Meeting minutes were written by KCR secretary and CLG staff took notes of proceedings; both documents were shared with the KCR groups in subsequent meetings.
8. Issues raised in meetings were forwarded to appropriate decision-making bodies within the research programme, feedback was provided at subsequent KCR meetings.

A survey conducted prior to KCR selection showed that one in eleven community members were in an active CBO.^23^ The name KCR was decided by the KCRs themselves, and their roles, which were predefined by KEMRI staff with external advisors, were deliberately NOT about proactive information giving or mobilisation on behalf of researchers. Instead their roles were to:

- reflect typical community views on the research centre's activities through regular feedback and *ad hoc* meetings between KEMRI staff and KCR members.
- increase community understanding of research through being able to respond to questions about KEMRI during their daily lives; and
- distribute Information Education and Communication (IEC) materials

It was clarified from the outset, including in the public endorsement meetings, that these roles were voluntary^24^ and were to be undertaken as part of KCR's normal daily life, that is, KCRs were not employees of the research programme. In addition to the quarterly meetings with a dedicated team of community facilitators employed within the community liaison group (CLG),^25^ KCRs could communicate with KEMRI through phone calls or text messages to a CLG hotline; letters by post; visits to the CLG office; or messages sent via staff members. A KCR guideline outlining selection processes and roles, methods of replacement of inactive members, and decision-making processes was developed and harmonized in consultation with all KCRs. In addition to T-shirts, programme support to KCRs included small travel and lunch allowances of Ksh.300 ($3.75)^26^ at each of 3 location-level meetings in a year, and a slightly higher amount for meetings at Kilifi; and an annual 2-day training workshop at Kilifi. Through these activities, there were opportunities to interact and co-learn among KCRs, and between KCRs, KEMRI and MoH staff.

## Methods

The community engagement activities at the KEMRI-Wellcome Trust Programme are an action research activity, approved by the national science and ethics committee of Kenya (SSC 1463).

In this paper we draw primarily on data from the KDHSS, on routine reports written by CLG staff or KCRs between 2005 and 2007, on self-administered questionnaires from KCRs and on focus group discussions with purposively selected KCRs. We also draw on CE routine reports to highlight changes taking place since this research was undertaken.

Records reviewed include minutes from KCR quarterly meetings (n = 56 reports), reports from training of KCRs (n = 7 reports), records of endorsement activities (n = 14), and community facilitator meeting minutes (n = 8). Self-administered questionnaires were filled by all 140 KCR members. DK interviewed KCRs selected on the basis of proportion of meetings attended; 6 KCR groups were selected to reflect varying attendance rates, and 4 members from each of those 6 groups interviewed (chairperson, secretary and two members). A total of three focus group discussions and 3 in-depth interviews were held with the KCRs.

KCR members gave verbal consent for use of routine data, and signed consent before participating in interviews. To ensure trustworthiness of findings given our involvement in the community engagement process, we followed accepted approaches for document analysis, involved more than one person in each step of the analysis, and held discussions on findings among ourselves and the wider CLG (see also Marsh et al., 2010).^27^

## Findings

### KCRs: characteristics and place of residence

**Figure 1:**
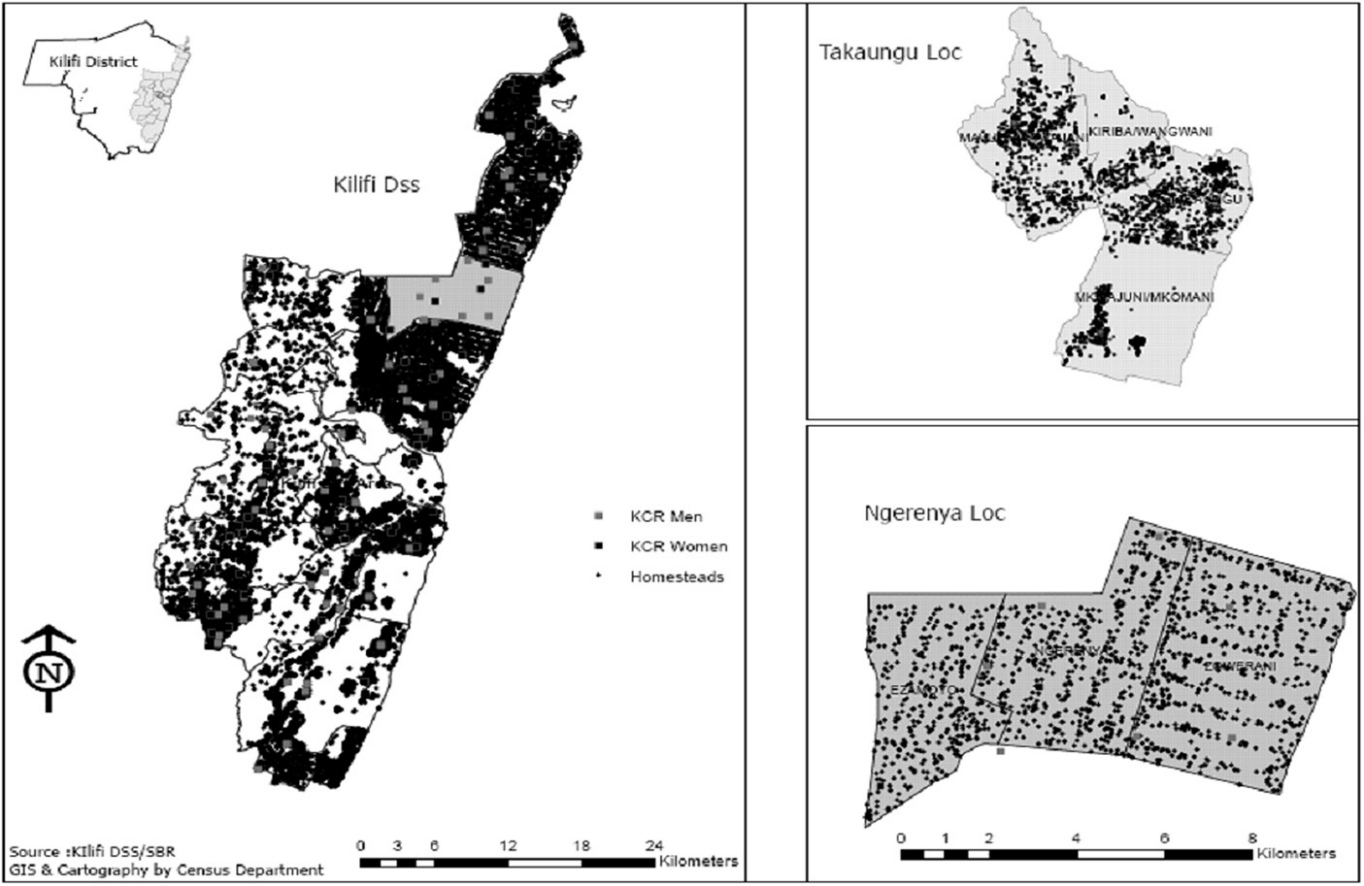
Spatial Distribution of KCRs across Kilifi HDSS

Overall, the gender distribution and proportion of main formal religions in KCRs was similar to that of the KHDSS population (Table 1). However KCR members were slightly older and more educated than the general population, and were less likely to report traditional beliefs. GPS data positioning of KCR residencies against population distribution in each location revealed that while KCRs were well distributed across locations, some poorly populated areas were not covered by a KCR (Figure 1). There was a wide range in the ratio of KCRs to the population per location (from 1:566 to 1:3016; Table 2), but these data are difficult to interpret: areas with low KCR/population ratios are often those with low population density.

**Table 1:** KCR and KHDSS population demographic characteristics

Attribute	Category	Population (n = 233,448)^1^ (%)	KCR (n = 141) (%)
Gender (%)	Female (%)	53	44
	Male (%)	47	56
Education (%)	*Proportion gone to school*	*42*	*100*
	Nursery	12	1
	Primary	78	36
	Secondary	8	54
	Post-secondary	2	5
	Missing data		3
Age	Proportion below 15 yrs	49	0
	15–19	23	1
	20–29	28	7
	30–39	19	34
	40–49	13	20
	50–59	9	22
	60–69	6	9
	70–79	3	1
Religion	Muslims	13	14
	Christians	47	83
	Traditional	24	0
	Other^2^	12	0
	Note reported	4	3

1 Source: 2005 Kilifi-DSS Census data.

2 Include Hindus, Budhism, Ba'arians.

**Table 2:** Ratio of KCRs to population and KCRs average meeting attendance

KCR Code	KCR members (n)	Locational population^1^	KCR: Population ratio	Ave^2^. Meeting attendance (5 meetings) (%)
01	8	9,342	1:1,038	67
02	10	17,543	1:1,754	84
03	6	6,106	1:1,018	90
04	8	4,532	1:566	61
05	9	27,147	1:3,016	62
06	8	4,730	1:591	73
07	16	41,150	1:2,572	70
08	10	13,250	1:1,325	76
09	10	13,784	1:1,378	86
10	10	14,895	1:1,490	72
11	10	10,222	1:1,278	76
12	14	27,003	1:1,929	83
13	11	21,417	1:1,947	79
14	10	10,715	1:1,191	62
15^3^		11,612		
***Total***	***140***	233,448	***1:1,585***	***74***

1 Sources: Kilifi-DSS census data, 2005.

2 Ave. = average.

3 Has no KCR since at the time of nominating KCRs, there were no KEMRI activities going on in the location apart from census.

**Figure 2:**
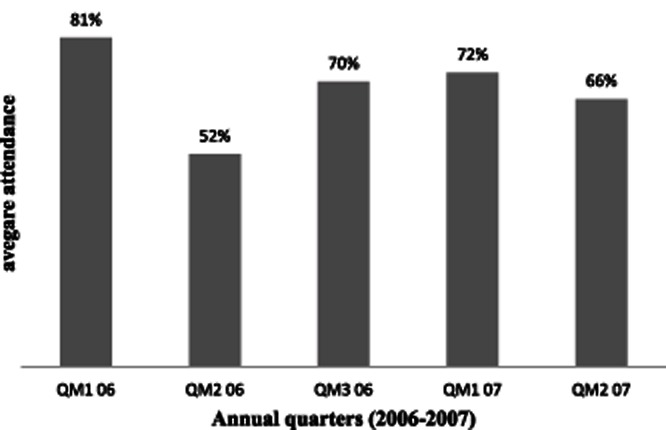
Proportion of KCR members attending quarter meetings (n = 140)

Average attendance for 5 meetings over an 18 month period for all KCRs was 74%, ranging from 30% (reported twice in two KCRs) to 100 % (reported 13 times in 7 KCRs) (Figure 2). Sixteen out of 140 KCR members (11%) ceased to be members over that time period. Of these, 3 died, 3 emigrated while 10 moved on to formal employment elsewhere. Eight of the 16 inactive KCRs (50%) had been replaced by July 2007 through direct election by community members in a public meeting organized and presided over by the area government administrator (chief).

The endorsement process of the KCRs suggested that the CBO approach to selection was more acceptable to community members attending public meetings than representatives being identified by chiefs. All KCRs nominated through CBOs were endorsed by the public, while three members of the three KCR groups nominated by Chiefs were rejected on the basis of non-residency and lack of transparency, and were later replaced with others directly elected by the community. A total of 140 KCR members were endorsed, chiefs and assistant chiefs (n = 50) were included as co-opted members in their respective locational KCR groups. As we have noted elsewhere, this suggests that public endorsement was an important check for fairness, accuracy and balance of individuals chosen to represent locations.^28^

### KCR descriptions of their roles and who they represent

KCRs generally described themselves as representing community views to KEMRI and KEMRI to the community, typically drawing the analogy of a bridge:

P5: … I am a community representative and I cannot represent the community without KEMRI. For example, if there is any concern in community, if the community members are not happy with the treatment given by KEMRI, I explain to KEMRI about that. At KEMRI, I get some knowledge, I assist the community members when they have concerns. … I therefore take concerns to KEMRI and some information from KEMRI to the community, *(male FGD2)*.

KCRs described their roles in a way that was consistent with those agreed at the outset (see above), reporting that their roles in CBOs and as KCRs were generally complementary, easily manageable and voluntary:

P1: … [you perform your roles] when you are doing your normal daily activities, not that you have to call a meeting, but in your daily activities that is when you do the roles. … *(female IDI)*

P1: … no one forces you, when you are going for these meetings that process of being forced to do something is not there … that is why I see it is not bad to continue being a KCR member … *(female, IDI)*.

However KCRs often described wanting to take on a more proactive role, including holding community outreach activities, accompanying field workers to homesteads, being informed of all studies and participants in a given area, and actively following-up research refusals to discuss the importance of research participation. The latter worried us because of the potential for KCRs to exert pressure on those unwilling to participate in research.^29^ Some KCRs requested roles that were not directly related to on-going research including dispersing over-the-counter drugs, selling and distributing bed nets and income-generating activities. These requests sometimes were a reflection of their own lack of clarity in the distinction between KEMRI as a health research organisation and as a treatment provider, and might also have been linked to community members' and their own priorities and concerns, and to KCR members wanting to consolidate their prior and increasing power positions and potential influence with regards to the research centre, as discussed below.

In one location, and repeated in other locations over time, some KCR members said that as a ‘bridge’ between KEMRI and the community, KCRs could also block information flow or distort information in situations where their individual requests were not met by KEMRI.

P2: … do you remember during the previous quarterly meeting where the chairman said, KCR *twaweza ganga na pia twaweza roga* [‘we can treat and we can bewitch’ meaning], we can say good things about KEMRI and at the same time say bad things about KEMRI … I mean with the influence KCR currently command in the community, we can decide to influence the community negatively if we want to, and they will listen *(male, FGD2)*

### Perceived benefits of being a KCR

The main benefits of being a KCR were reported to be an increased knowledge of medical research and of KEMRI's role, and having gained some information on health issues. Also mentioned as benefits were the various types of support offered to KCRs described above, including t-shirts and travel and lunch allowances. Many KCRs also reported that being a KCR strengthened their prominence and respect at the interface: i.e. both in the community and with KEMRI staff (Box 2, quotes 1 and 2). There were hints throughout discussions that a strengthened relationship with KEMRI was expected to be reciprocal; that while they act as a bridge to the community, KEMRI should also be a bridge to improved treatment for themselves or their families (Box 2; quote 3).

**Box 2. Illustrative quotes – Benefits and challenges of being a KCR**

1^st^ quote

… Just to add, I thought it [being a KCR member] develops a sense of respect and I want to retain that respect not to let my community members down **(P1, FGD2)**

2^nd^ quote

… It is due to the good relationship between KCR and CLG …… If the CLG office was frustrating us, we would have left long time ago **(P2, FGD2)**

3^rd^ quote

… I think that I will be attended as one of them (KEMRI staff). I would tell them to investigate me (*nichunguzini*). I feel this and that. It would be easy for me to say because there is that freedom of communication. It is not the same as someone who has never known you, never seen you, has only been written for a name, you will then say we do not do those investigations, go to (Kilifi) hospital … I will then get support, and you will be like you have gotten a stepping stone **(P1, female, IDI).**

4^th^ quote

… people think that if they go to them [KCR] they will get a chance to go direct to KEMRI. The community members think that KCRs are doors to KEMRI but they don't know that KEMRI aims to benefit the whole community … some community members follow them [KCRs] for drugs and the KCRs have none. **(CH2, QM 06, CFN (community facilitator notes)).**

5^th^ quote

… the community are complaining to him [KCR] that ‘we endorsed you to educate us about KEMRI and research but you ain't visiting us to do so. We will not accept you come next election. They compare us with a local (name) CBO members who visit households to educate them on various issues of their concern. So, he said the community have high expectations from them … **(TK, 2QM 07).**

6^th^ quote

… People in the Matatu [public minibus] started talking about KEMRI, … two KCR members from Jaribuni and later [another KCR], tried to explain the role of KEMRI, and were told that they had been induced into devil-worship, that is why they were defending KEMRI. … other KCR members said it is common in the community for people to say KEMRI is devil worship organization. … [because] of the big vehicles, big salaries, many workers, and they ask where KEMRI gets all the money from **(JB1, QM 06,CFN)**

Certainly, there was a constant series of requests from KCRs for employment, funds, treatment services, health advice, transport lifts and various other resources. Some requests took a substantial amount of time in negotiation. For example ‘bus fare’ refunds were initially agreed at a rate of $2 for scheduled quarterly locational meetings; an amount that was above the average daily income of less than $1 in rural areas and $2 in urban centres at the time. The amount was doubled following discussions, but immediately thereafter discussions began for a higher rate. Employment into the research programme is generally viewed as a main benefit to the community, with requests for employment opportunities for KCR members, their relatives and members of the broader community made regularly and strongly in KCR meetings.

### Perceived challenges of working at the interface

An important challenge raised repeatedly by all KCRs concerned the frustration that arose as a result of community members generally not having a clear understanding of their roles, with the perception that KCRs could help them access treatment from KEMRI, or that they should be more proactive in engaging with community members (Box 2; quotes 4 and 5). In responding to concerns about KEMRI, especially around rumours about devil worship that are typical of our setting and many others,^30^ some community members accused KCRs of having ‘sold out’ to the organisation in order to gain large allowances (Box 2; quote 6). Other more practical challenges for KCRs included long distances between each KCRs residence making meetings difficult, not having any resources to organise their own meetings outside the quarterly meetings, and a sense of having been given an overwhelming amount of information about studies.

### KCRs reports of their impact

Despite some of the above challenges, KCRs described their impact on the community in dramatically positive terms, including for example the community having a better understanding of research, improved health, reduced mortality and being treated better in the Kilifi District Hospital. They also described a reduction in rumours and concerns about KEMRI, and reported that KEMRI is now more accepted in the community:

P2: … this has also changed the picture of KEMRI, because we are respected and we are associated with KEMRI, then KEMRI is also being looked at as a respectable organization. So the bad picture of KEMRI is changing, *(male, FGD2)*.

Interview bias is expected to have influenced the above reports and comments, but of interest is that the issues that were raised and discussed in KCR meetings (discussed above as personal KCR requests, and also the more research related comments summarised in Box 2) are reflective of issues raised through more in-depth discussions with a range of community members,^31^ suggesting that the concerns brought forward in meetings are in some way ‘representative’ of many others living in the area.

### Contributions of KCRs to changing policies at the programme

While research staff might not express the impact of the KCR network in such dramatic terms, issues raised through the network have contributed to a whole series of policy and practice changes within the unit including new more transparent employment policies focusing on local employees from across the KDHSS wherever possible,^32^ a new schools-based participatory action research project aimed at increasing exposure of science students in local schools to researchers and research activities as regularly requested by KCRs,^33^ new guidelines concerning community engagement for all studies at the programme aimed at meeting demands for more information-sharing on the research programme and studies,^34^ and steps towards the development of institutional guidelines for the research programme regarding benefits and payments.^35^ More broadly, the increasing amount of interaction between KEMRI staff and community members through this network is perceived by the community liaison group to have strengthened mutual understanding and trust.^36^

## Discussion

Key challenges in community engagement include the definition of communities, and who can be considered to authentically represent those communities.^37^ In Kilifi, we established a network of representatives of a geographical area covering approximately 240,000 people living in the locations surrounding Kilifi district hospital, as one mechanism to interact more with community members. The intention was both to enable residents of the locations to assist with the selection of representatives, and to ensure that those representatives come from and were aware of ideas and concerns across the area.

In this paper, we have shown that the KCR members selected using the combination of approaches described led to community members being representative of the general community in terms of gender, but being slightly older and better educated, and less likely to follow traditional beliefs. The network was well sustained over the 18 months we analysed, with a high degree of meeting attendance and low degree of turnover rates. We have also shown that the KCR members reported good understanding of their roles, suggesting good training, and that they raised many issues either personally or on behalf of community members that we understand are ‘typical’ of the priorities and concerns of many community members, including an interest in and demand for more personal benefits in terms of access to health care and other resources. A clear term for each KCR network of two to three years enables the amount of social interactions and potential mutual understanding through open discussion and critique to increase significantly over this period of time.

The issues raised through the network have contributed to a series of policies and activities that have important implications for how studies are designed and implemented on the ground, including how communities are consulted and informed about studies, new thinking about appropriate benefit sharing approaches across the programme, more locally appropriate consent processes, and fairer employment policies. These changes are all aimed at ensuring that in contexts where research is much needed but strong inequities and potential for unfairness exist, ethical practice is supported not only through careful prior review that includes an awareness of local priorities and concerns, but also by careful consideration of and monitoring of how research is actually being conducted on the ground, over the entire course of studies. Of interest was that these achievements were not clearly articulated by KCRs themselves. Ensuring that these contributions are reported back to network members over time could contribute to greater motivation and mutual understanding, including the very real contributions, and their limits, that this network of representatives can and should make. The latter is not straightforward, given the range of influences on policy, practice and experience in such a large and complex research institution, and the range of different types of representatives engaged with in addition to the KCR network.

There are several more specific concerns regarding the functioning of this initial network of KCRs. Firstly, although relatively well spread out geographically, the KCRs are not entirely typical of the general community, in that the most vulnerable groups are least likely to be involved in community-based organisations, or to be nominated from within CBOs to attend open days at the programme and be selected and endorsed as locational representatives. In the subsequent rounds of KCRs, in an effort to reduce this concern and to strengthen geographical representation, selection was not through CBOs but through public meetings at the sub-locational level (43 meetings, with an average attendance of 250 people (range 80–400). 172 KCR members were elected, 85 (50%) of whom were men, and a further 53 administrative leaders co-opted to KCRs. Although not yet formally analysed, this approach is understood by the community liaison team to have ensured that KCRs are more typical of other community members in terms of levels of literacy and religious beliefs. Given that many social networks in this community, as in others, are horizontal rather than vertical (i.e. with others with similar characteristics and levels of resources),^38^ issues raised by members of the network are likely to be representative of others in the community, in turn strengthening mutual understanding. Nevertheless, we recognise that the most vulnerable groups in terms of income and literacy levels in the community will remain underrepresented in this network; an important bias given that many research participants in our setting have very low literacy levels.^39^ This point illustrates the importance of ensuring that such a network is continually amended to strengthen representation of all community members, and that it is at most only one part of a wider community engagement strategy for a research programme and study, which might need to seek out views and concerns of specific vulnerable groups in other ways (for example through specifically designed qualitative studies or consultations with target groups). With regards to the new KCR network, election by residents in the sub-location in which they live presents not only possibilities (for example, of building relationships and a sense of collaboration between communities and the programme), but also challenges, such as expectations among community members that KCRs should be lobbying for greater research-related benefits for their own location, and a possibility of KCRs being perceived as having ‘failed’ if they do not do this.

Another potential concern is the amount of discussion in KCR meetings about personal allowances and benefits, and in particular access to treatment. Although these discussions can be frustrating for everybody involved, we believe that these might be an inevitable part of building a relationship and mutual understanding between a relatively well resourced research institution, and representatives of a low income community with inadequate health care. As described elsewhere,^40^ discussions around personal benefits might be linked to the relatively vague distinction between voluntary activity and employment, and descriptions by researchers of payments as reimbursements of cost incurred and of time, while they are perceived as simple payments by KCRs and community members.^41^ One issue with regards to levels of payment, as also discussed by Angwenyi et al. in this collection is that extrinsic motivations such as cash have the potential to crowd out intrinsic motivations, such as interest in protection of communities. Payments may also introduce relationship challenges between representatives and their community members. On the other hand where there is no motivation, or there are costs to volunteers, there is a possibility that the goals of community engagement (including to strengthen research relationships and ethical practice) will be undermined. The negotiations concerning the levels and types of reimbursement and motivation, if handled well, may represent a process of building trust within the relationship^42^ where each agreement is tried, tested and re-evaluated. Community representatives begin to better understand the funding constraints and limitations of a particular study or of the institution, while the research organisation learns about areas that may need greater flexibility or focus in funding, or greater explanation. With the second network of KCRs these negotiations have been less regular and emotional, as the terms were clearer and more appropriate at the outset, but they remain present. Discussions with KCRs have contributed to an interest in developing a clearer policy on benefits for individuals and participants for all the studies,^43^ and once the studies are over. With regard to the specific issue of access to relatively good treatment through KEMRI, there is clearly a need to continue to explain in community engagement activities that the support that KEMRI provides for in-patient paediatric services through the district hospital is available to all children, regardless of participation in research.

A related concern to the negotiation of additional personal benefits is the request from KCRs for more proactive roles (which would change the role of KCRs closer to staff, and require some form of remuneration). There were a number of reasons for the limited role for KCRs, including the complexity of many research messages and the difficulty of knowing if and how these are passed on to others, and – most importantly – an interest in ensuring that KCRs remain able to independently critique KEMRI. This is clearly a very difficult balance to get right: to adequately compensate for time spent with KEMRI on meetings, and to avoid perceptions or realities of the network representing KEMRI rather than the community. It is a balance that is likely to be a challenge in many contexts, and one that we in Kilifi aim to continue learning about and monitoring over time.

## Conclusion

Community engagement is increasingly advocated in international biomedical research for both intrinsic ethical values (for example, showing respect) and for instrumental purposes (for example, improving consent processes). The KCR network in Kilifi is aimed at geographical representation, with members being typical of the areas that they come from, and with residents from that area having some say in their selection. We have found this approach a valuable way of strengthening the relationship between researchers and local populations, with the potential to build trust and counter concerns and rumours, and ultimately to strengthen ethical practice. Strong support from senior leaders at the research programme has been essential; as has adequate long term funding.

There are however clear challenges and limitations with the functioning of this network. These challenges highlight the importance of continuous discussion about and clarification of roles, payments and lengths of time of service; to the need to respond to diverse problems when they arise through a well organised and supported community liaison group; and the potential for inadequate inclusion and therefore direct representation of the most vulnerable and marginalized members in the network. Furthermore, KCRs have the potential to act in a way that is counter to community engagement goals by adopting gate-keeper roles or by perceiving themselves as increasingly accountable to researchers as opposed to community members. Finding other ways to achieve deeper and broader levels of community engagement have therefore been, and remain, essential in our setting. Given the growing interest in, and in some cases, requirement for Community Advisory Boards or Groups (CAB/ CAGs) by funders and ethics committees, and that our findings resonate clearly with the strengths and challenges documented by others working with representative groups and networks, our research experiences are likely to continue to draw upon and be relevant to others working at the interface between research institutions and communities in similar low income settings.

## Footnotes

1 D.A. Diallo, et al. Community permission for medical research in developing countries. *Clin Infect Dis* 2005; 41: 255–259, O.K. Doumbo. Global voices of science. It takes a village: medical research and ethics in Mali. *Science* 2005; 307: 679–681.

2 C. Weijer & E.J. Emanuel. Ethics. Protecting communities in biomedical research. *Science* 2000; 289: 1142–1144, L.W. Green & S.L. Mercer. Can public health researchers and agencies reconcile the push from funding bodies and the pull from communities? *Am J Public Health* 2001; 91: 1926–1929.

3 A. Nyika. Ethical and practical challenges surrounding genetic and genomic research in developing countries. *Acta Trop* 2009; 112 Suppl 1: S21–S31, F. Tekola, et al. Impact of social stigma on the process of obtaining informed consent for genetic research on podoconiosis: a qualitative study. *BMC Med Ethics* 2009; 10: 13.

4 R.R. Sharp & M.W. Foster. Grappling with groups: protecting collective interests in biomedical research. *J Med Philos* 2007; 32: 321–327, L.F. Ross. 360 Degrees of human subjects protections in community-engaged research. *Sci Transl Med* 2010; 2: 45cm23, L.F. Ross, et al. Nine key functions for a human subjects protection program for community-engaged research: points to consider. *J Empir Res Hum Res Ethics* 2010; 5: 33–47, L.F. Ross, et al. Nine key functions for a human subjects protection program for community-engaged research: points to consider. *J Empir Res Hum Res Ethics* 2010; 5: 33–47.

5 R.P. Strauss, et al. The role of community advisory boards: involving communities in the informed consent process. *Am J Public Health* 2001; 91: 1938–1943.

6 Diallo, et al., *op.cit* note 1, Doumbo et al., *op.cit* note 1.

7 P. Reddy, et al. The role of community advisory boards in health research: Divergent views in the South African experience. *SAHARA J* 2010; 7: 2–8.

8 K. Shubis, et al. Challenges of establishing a Community Advisory Board (CAB) in a low-income, low-resource setting: experiences from Bagamoyo, Tanzania. *Health Res Policy Syst* 2009; 7: 16, S.F. Morin, et al. Building community partnerships: case studies of Community Advisory Boards at research sites in Peru, Zimbabwe, and Thailand. *Clin Trials* 2008; 5: 147–156, Sharp & Foster *op.cit* note 4.

9 D. Brieland. Community advisory boards and maximum feasible participation. *Am J Public Health* 1971; 61: 292–296, Morin et al., *op.cit* note 8; Sharp & Foster *op.cit* note 4; Shubis et al., *op.cit* note 9.

10 D.F. Ragin, et al. Defining the ‘community’ in community consultation for emergency research: findings from the community VOICES study. *Soc Sci Med* 2008; 66: 1379–1392.

11 V.M. Marsh, et al. Working with Concepts: The Role of Community in International Collaborative Biomedical Research. *Public Health Ethics* 2011; 4: 26–39.

12 Reddy et al., *op. cit* note 7, Shubis et al., *op.cit* note 8.

13 Shubis et al., *op.cit* note 8.

14 Morin et al., *op.cit* note 9.

15 C. Molyneux, et al. The role of community-based organizations in household ability to pay for health care in Kilifi District, Kenya. *Health Policy Plan* 2007; 22: 381–392.

16 Reddy et al., *op. cit* note 7; Shubis et al., *op.cit* note 8; Morin et al., *op.cit* note 9.

17 Reddy et al., *op.cit* note 7.

18 V. Marsh, et al. Beginning community engagement at a busy biomedical research programme: experiences from the KEMRI CGMRC-Wellcome Trust Research Programme, Kilifi, Kenya. *Soc Sci Med* 2008; 67: 721–733.

19 CGMRC – Centre for Geographic Medicine Research Centre.

20 J.A. Scott, et al. Profile: The Kilifi Health and Demographic Surveillance System (KHDSS). *Int J Epidemiol* 2012.

21 Marsh et al., *op.cit* note 18.

22 CBOs included formally registered groups such as land-buying groups, farmer groups, development groups, as well as small scale informal groups such as merry-go-rounds (cash transfer groups), small traders, and youth groups. We identified 569 groups, one-third of which were unregistered.

23 Marsh et al. *op.cit* note 18.

24 KCR members were reimbursed for expenses, and were not paid a stipend for being a KCR member.

25 Dedicated team of 10 KEMRI employees backstopped by 2 senior researchers; trained on facilitation and communication skills and research ethics. Their primary responsibility is coordinating programme-wide and study-specific community engagement activities.

26 Exchange rate at the time: $1- =- Ksh. 75 in 2007.

27 V.M. Marsh, et al. Experiences with community engagement and informed consent in a genetic cohort study of severe childhood diseases in Kenya. *BMC Med Ethics* 2010; 11: 13.

28 V. Marsh, et al., *op.cit* note 18.

29 S. Molyneux, et al. Community members employed on research projects face crucial, often under-recognized, ethical dilemmas. *Am J Bioeth* 2010; 10: 24–26.

30 C.S. Molyneux, et al. Understanding of informed consent in a low-income setting: three case studies from the Kenyan Coast. *Soc Sci Med* 2004; 59: 2547–2559, P.W. Geissler & R. Pool. Editorial: Popular concerns about medical research projects in sub-Saharan Africa – a critical voice in debates about medical research ethics. *Trop Med Int Health* 2006; 11: 975–982, C. Gikonyo, et al. Taking social relationships seriously: lessons learned from the informed consent practices of a vaccine trial on the Kenyan Coast. *Soc Sci Med* 2008; 67: 708–720.

31 Gikonyo et al., *op.cit* note 30; Marsh et al., *op.cit* note 18; Marsh et al., *op.cit* note 11.

32 D.M. Kamuya, et al. Evolving Friendships and Shifting Ethical Dilemmas: Fieldworkers' Experiences in a Short Term Community Based Study. *Developing World Bioethics* 2012; in press.

33 A. Davies, et al. Seeing ‘With my Own Eyes’: Strengthening Interactions between Researchers and Schools*. *IDS Bulletin* 2012; 43: 61–67.

34 Institutional community engagement guidelines are available from the authors, as are consent templates and consent SOP guidelines. The latter have drawn on ideas and concerns raised by KCRs as well as other community engagement activities at the programme.

35 S. Molyneux, et al. Benefits and payments for research participants: Experiences and views from a research centre on the Kenyan coast. *BMC Med Ethics* 2012; 13: 13.

36 Marsh et al. op.cit note 19.

37 C. Rotimi, et al. Community engagement and informed consent in the International HapMap project. *Community Genet* 2007; 10: 186–198.

38 C. Molyneux, et al. The role of community-based organizations in household ability to pay for health care in Kilifi District, Kenya. *Health Policy Plan* 2007; 22: 381–392.

39 Molyneux et al., *op.cit* note 30.

40 P.W. Geissler. ‘Transport to Where?’. *Journal of Cultural Economy* 2011; 4: 45–64.

41 Ibid.

42 Gikonyo et al., *op.cit* note 30.

43 Geissler Molyneux et al., *op.cit* note 38; V.M. Marsh, et al. Working with Concepts: The Role of Community in International Collaborative Biomedical Research. *Public Health Ethics* 2011; 4: 26–39.

